# Factors associated with fear and coping strategies during pandemic in female university students: The moderator role of physical activity and infection status

**DOI:** 10.1371/journal.pone.0355544

**Published:** 2026-08-11

**Authors:** Emre Karaduman, Nazlıcan Toprak, Adem Çevik, Serhat Erail, Muhammet Hakan Mayda, Aydan Ermiş, Kürşat Acar, Kenan Şebin, Menderes Kabadayı, Ali Kerim Yılmaz, Ayşe Anderiman, Eda Karaduman, Özgür Bostancı

**Affiliations:** 1 Faculty of Sport Sciences, University of Ondokuz Mayıs, Samsun, Türkiye; 2 Faculty of Sport Sciences, University of Kilis 7 Aralık, Kilis, Türkiye; 3 Faculty of Sport Sciences, University of Sinop, Sinop, Türkiye; 4 Faculty of Sport Sciences, University of Atatürk, Erzurum, Türkiye; Soochow University, CHINA

## Abstract

The COVID-19 pandemic has generated widespread psychological distress worldwide, with fear emerging as a central emotional response; however, its role in shaping coping processes, particularly among female university students, remains unclear. The present study investigates the relationship between fear of COVID-19 and coping strategies in female university students, with a specific focus on the moderating role of various variables. Participants were 1000 female university students aged 21.5 (2.5) years, who completed a self-report questionnaire about levels of fear regarding COVID-19 and the coping strategies employed during the post-emergency phase. The result showed that fear of COVID-19 (FCV-19S) was positively related to coping with the outbreak (COS). Physical activity status and COVID-19 positive or negative experiences moderated the relationship between FCV-19S and COS. These findings suggest that individual characteristics such as sedentary lifestyle habits and firsthand infection experiences, may continue to shape COVID-19-related fear and coping responses even after the acute emergency phase has subsided. An intervention such as regular exercise that aims to develop and improve coping dynamics among people in response to new challenges posed by stressful events or public health crises, like COVID-19 infection, seems warranted.

## Introduction

The COVID-19 pandemic has caused unprecedented global health concerns, not only affecting individuals’ physical health but also often directly their mental health [1–3], with several variants still continuing to pose threats. Among these concerns, a sense of fear, especially fear of COVID-19, emerged as a prevailing and dominant experience for young college students [4]. The virus’s high potential for contagion, serious health problems, and lethal nature have instilled a fear in individuals, making them feel that both themselves and their loved ones are at risk [1,5,6].

Fear, as a primal emotion, triggers various psychological and physiological reactions that can result in maladaptive behaviors or elevated stress responses detrimental to one’s health [7,8]. Previous studies have shown that fear in the pandemic context can lead to serious stress related mental consequences [9–11]. A systematic review of 398,771 participants reported the prevalence of psychological problems such as depression, anxiety, and distress during the COVID-19 pandemic were 28%, 27%, and 50%, respectively [12], while a meta-analysis of 221,970 participants found the pooled prevalence rates for these disorders were 31%, 32%, and 41%, noting a marked rise since the pandemic’s onset [13]. Given the numerous psychological challenges that COVID-19 presents, it is crucial to comprehend the factors linked to fear and the subsequent coping strategies adopted by individuals.

Coping strategies, which are typically accepted as cognitive and behavioral efforts to manage specific internal and external demands [14], have emerged as one of the most important tools people have used to reduce the psychological and physiological harms associated with the threat of infection during the pandemic [15]. Considering the symbiotic relationship between fear and coping, it can be inferred that the magnitude of fear resulting from individual differences might dictate the selection and efficacy of coping strategies, and inversely, the efficacy of a coping strategy can modulate the levels of fear [14]. Therefore, the theoretical framework of the study was constructed based on the Stress and Coping Process Model proposed by Lazarus and Folkman [14]. This model suggests that individuals’ responses to stress are shaped by their cognitive appraisal of the stressor (e.g., fear of COVID-19) and the coping resources they can utilize. Fear of COVID-19, conceptualized as a perceived environmental threat, can trigger coping strategies at varying levels depending on personal characteristics (e.g., individual experiences) [9].

Previous studies have reported that young female students may be susceptible to mental health issues [16,17] and the development of different coping strategies [18]. Thus, it is essential to note that fear and coping strategies may vary across different populations. In particular, it is stated that the interactions between biological factors, social determinants, gender stereotypes, gender roles, social stigma, inequality, and social autonomy contribute to women experiencing more stress, anxiety, and depression than men [19–21]. This gender factor emerges as an important determinant in both the experience of fear and the development of coping strategies during or post-pandemic. Previous studies have suggested that both adult and college-aged women are affected by the adverse psychological impacts of the pandemic due to increased fear [4,6,22,23]. Despite the growing body of research examining the psychological consequences of the COVID-19 pandemic, the factors that may influence the relationship between fear of COVID-19 and coping strategies remain insufficiently understood, particularly among female university students. Existing studies have primarily focused on the independent effects of fear or coping outcomes, whereas evidence regarding individual characteristics that may modify this relationship is limited. Therefore, the present study contributes to the literature by exploring the potential moderating roles of physical activity status and COVID-19 infection experience in a large sample of female university students, providing a more comprehensive understanding of factors associated with adaptive responses during public health crises.

**Fig 1:**
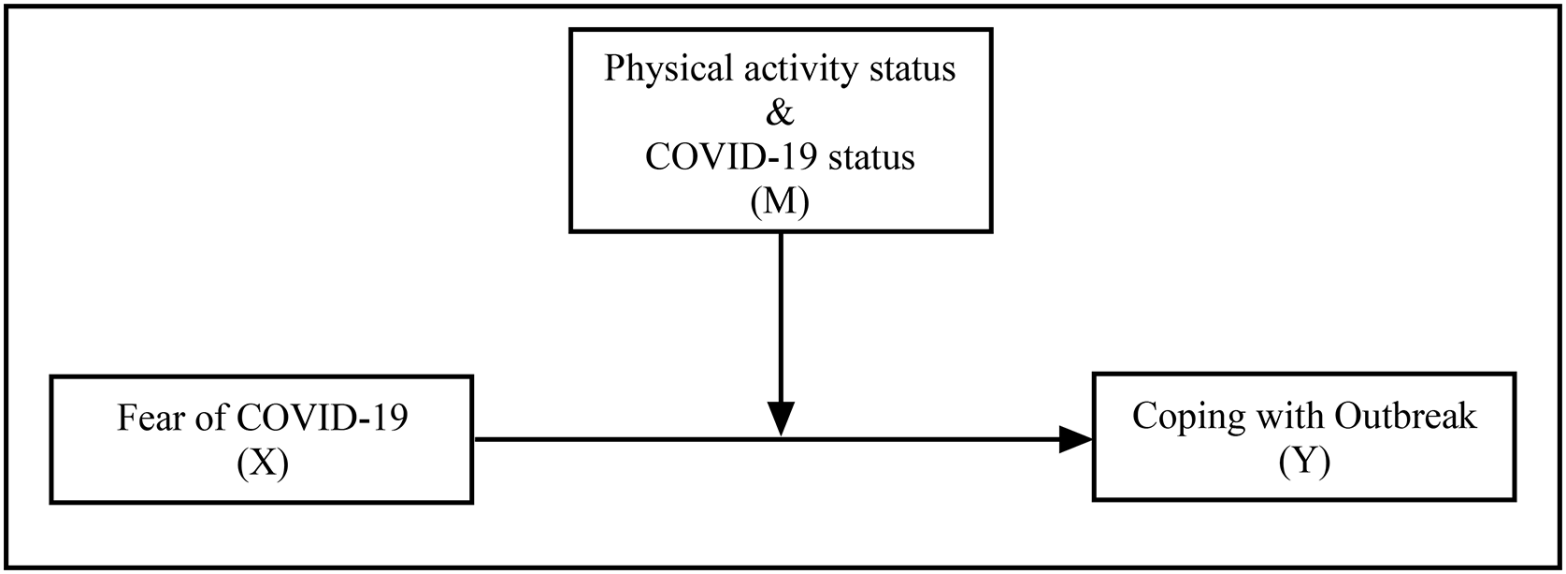
Conceptual diagram testing the moderation of variables between FCV-19S and COS.

Therefore, the main objective of this study was to investigate the moderating effect of various variables on the relationship between fear of COVID-19 and coping strategies utilized by female university students during the post-emergency phase. First, we hypothesized that there is a significant relationship between COVID-19 fear and coping strategies in female university students (H_1_). Second, we hypothesized that various factors may have moderating effects of COVID-19 fear on strategies to cope with the outbreak (H_2_). Based on previous research, a research model was developed to test the following hypothesis (Fig 1).

## Materials and methods

### Participants and procedure

The sample includes students from various faculties at the University of Ondokuz Mayıs in Türkiye, including Dentistry, Divinity, Education, Health Sciences, and Medicine. This study employed a convenience sampling approach to recruit 1000 female university students. This method was chosen due to its practicality in accessing participants within the researchers’ professional networks, as well as the significant restrictions commonly encountered in scientific research conducted during the post-emergency phase. Among participants classified as COVID-19 positive, infection status was based on self-reported laboratory-confirmed diagnoses recorded in the official Turkish Ministry of Health electronic health record system (e-Nabız), which is directly accessible to individuals. Furthermore, all participants with a COVID-19 infection reported having received vaccination prior to infection. The recreationally active group comprised individuals who declared dedicating more than six hours per week to activities such as running, cycling, swimming, tennis, or fitness. On the other hand, the sedentary group consisted of females who did not regularly practice any sports activities or did less than two hours of exercise per week.

A cross-sectional research design was employed in which two standardized scales were used, and their associations with various variables were examined. In this study, the fear was assessed by the Fear of COVID-19 scale (FCV-19S) [11], and coping strategies were evaluated by the Coping with Outbreak Scale (COS) [24]. Participants were required to: a) be at least 18 years of age, b) have no mental or neurological disorders, c) be female university students, and d) consent to participate. Eligibility criteria have been kept to a minimum to ensure the inclusion of the target individual. However, the study excluded 119 students who still did not meet the conditions above and did not fully respond to all of the survey questions. The data of the study were collected via face-to-face administration of a questionnaire from willing volunteers between July-December 2023. The personal information form and scales took to complete 7−10 minutes. The name and other personal information of the study participants were protected. This study was conducted in accordance with the Declaration of Helsinki and was approved by the Social and Human Sciences Ethics Committee at the University of Ondokuz Mayıs (approval number: 12901−562). All participants provided written informed consent.

### Measures

#### The personal information form.

This form was used to collect data on the participants’ socio-demographic information, including age, faculty, physical activity, and COVID-19 positive and negative status (oneself or close circles). Additional information was also collected regarding participants’ COVID-19 vaccination status and number of vaccine doses received. Furthermore, the collected data includes various aspects such as the severity of the disease in those who have had COVID-19, confirmed infection, or death (from COVID-19 infection) information in relatives or close friends. In the present study, the FCV-19S and COS were used to evaluate participants’ current COVID-19-related responses during the post-emergency phase.

#### Fear of COVID-19 Scale (FCV-19S).

This scale was developed by Ahorsu et al. [11] and subsequently adapted to Turkish by Satici et al. [25]. The scale consists of a unidimensional, seven item, and five-point Likert-type scoring system (strongly disagree = 1 to strongly agree = 5). Factor loadings of the original scale range from .66 to .74, and corrected item-total correlations vary between .47 and .56. The internal consistency (α = .82) and the scale’s test-retest reliability (ICC = .72) were acceptable. Furthermore, the FCV-19S was positively correlated with perceived vulnerability, hospital anxiety, and depression [11]. In the present study, Cronbach’s α for the scale was .85. There is no reverse item on the scale. The total score of the fear of COVID-19 scale, obtained by summing the individual response items, ranges from 7 to 35, with higher scores indicating greater fear of COVID-19.

#### Coping with the outbreak scale (COS).

The Coping with Outbreak Scale (COS) was developed by Hatun et al. to reveal coping strategies during the COVID-19 outbreak [24]. The scale includes a four-teen item and structurally six-point Likert-type scoring system (I have never done it = 0 to I have done it very often = 5). The scale includes four dimensions: cognitive, transcendental, behavioral, and relational coping. In participants’ coping strategies during the post-emergency phase, the cognitive dimension involves accessing accurate information and self-suggestion, the transcendental dimension entails resorting to hopeful and spiritual means, the behavioral dimension includes acquiring various occupations, and the relational dimension involves seeking social support. The factor loading of the original scale was .84, and the corrected item-total correlations ranged between .39 and .71. The reliability coefficients for each sub-dimension are as follows: cognitive coping (.77), transcendental coping (.70), behavioral coping (.76), and relational coping (.79) [24]. In the present study, the Cronbach’s alpha coefficient was .88 for the total scale and .79, .78, .78, and .83 for the cognitive, transcendental, behavioral, and relational coping strategies, respectively. There is no reverse item on the scale. A high score on the scale indicates that the person has a high coping ability against the pandemic.

### Data analysis

Data analyses were conducted using IBM SPSS Statistics version 25.0 (IBM Inc., Armonk, NY, USA). Descriptive data were presented as mean, standard deviations (SD) and frequencies (%). Normality assumption was evaluated by visual inspection of the histograms (approximating a normal distribution) and applying a Kolmogorov–Smirnov test. The relationships between the FCV-19S and the COS were evaluated with Spearman’s rho (ρ). A correlation of ≥0.10 was considered as small, ≥ 0.30 as moderate, and ≥0.50 as large in terms of effect size. The moderation analysis was conducted using Model 1 of the PROCESS Macro (version 4.3) integrated with SPSS [26] to test the moderating effects of variables (M) between the FCV-19S (X) and the COS (Y) (Fig 1). The statistical significance of the moderation was set at 95% confidence intervals (CI) with the recommended 5000 bootstrap samples [27]. An interaction effect was supported by a significant R^2^ change (ΔR^2^). Aguinis suggests that interaction effects are expected to explain an additional range of 1% to 3% in unique variability related to the criterion [28]. We also reported unstandardized coefficients (*B*) for moderation analyses. Graphical tools were utilized to show the slope of the moderation outcomes (Fig 3). Furthermore, we used Harmon’s one-factor test to assess common method bias. If the percentage variance extracted of the single factor over 50%, the common method biases issue was detected. The Harman’s single-factor results demonstrated the single factor value at 28.4% of the total variance. This guaranteed the common method bias did not appear in this study. Graphical representations were generated using GraphPad Prism (Version 10; GraphPad Software, San Diego, CA, USA, http://www.graphpad.com/).

**Fig 2:**
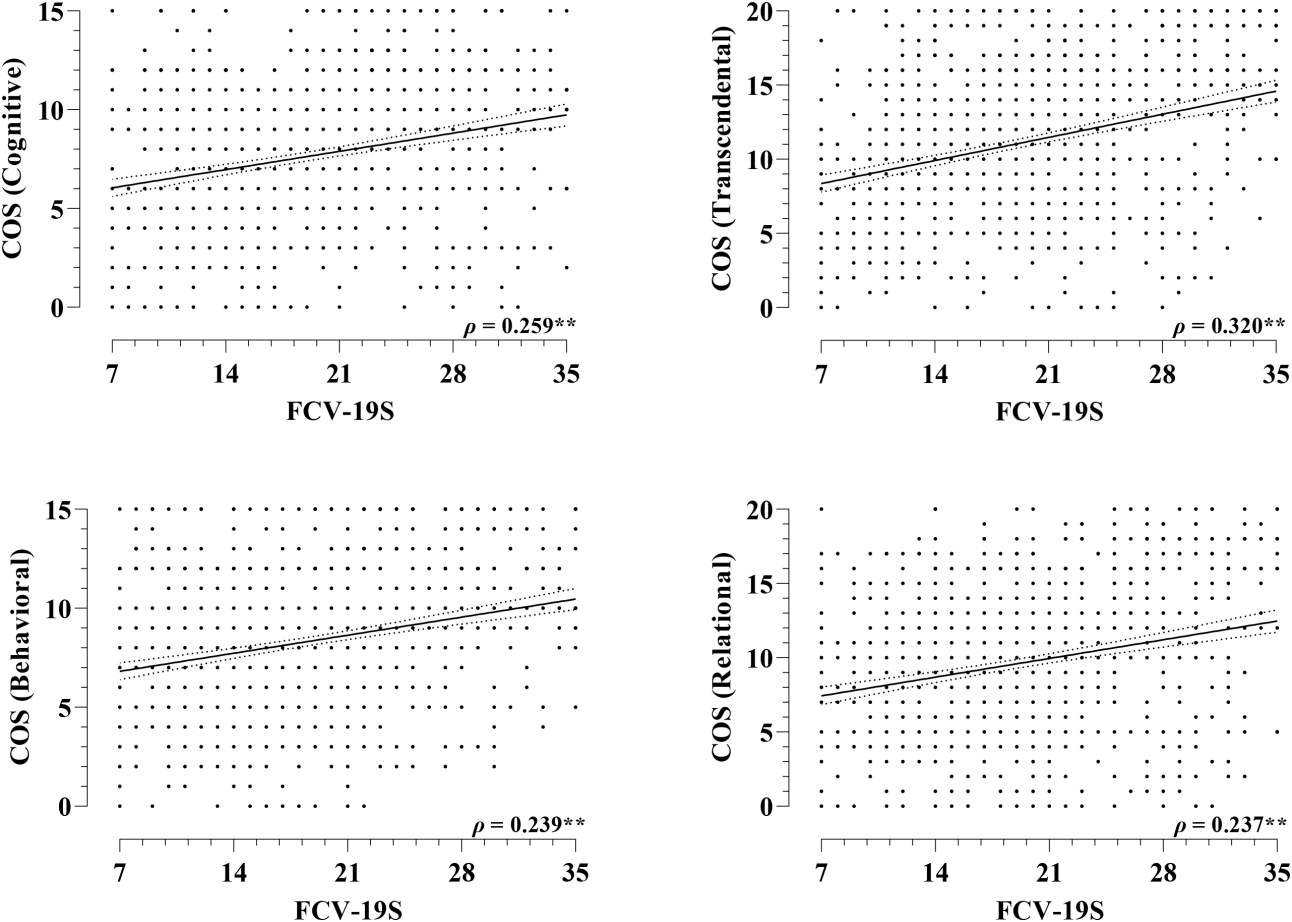
Scatterplots with regression lines and correlation coefficients between FCV-19S and COS. COS = Coping with Outbreak Scale; FCV-19S = Fear of COVID-19 Scale; ** p < 0.001. Data are presented as correlation coefficients (ρ).

**Fig 3:**
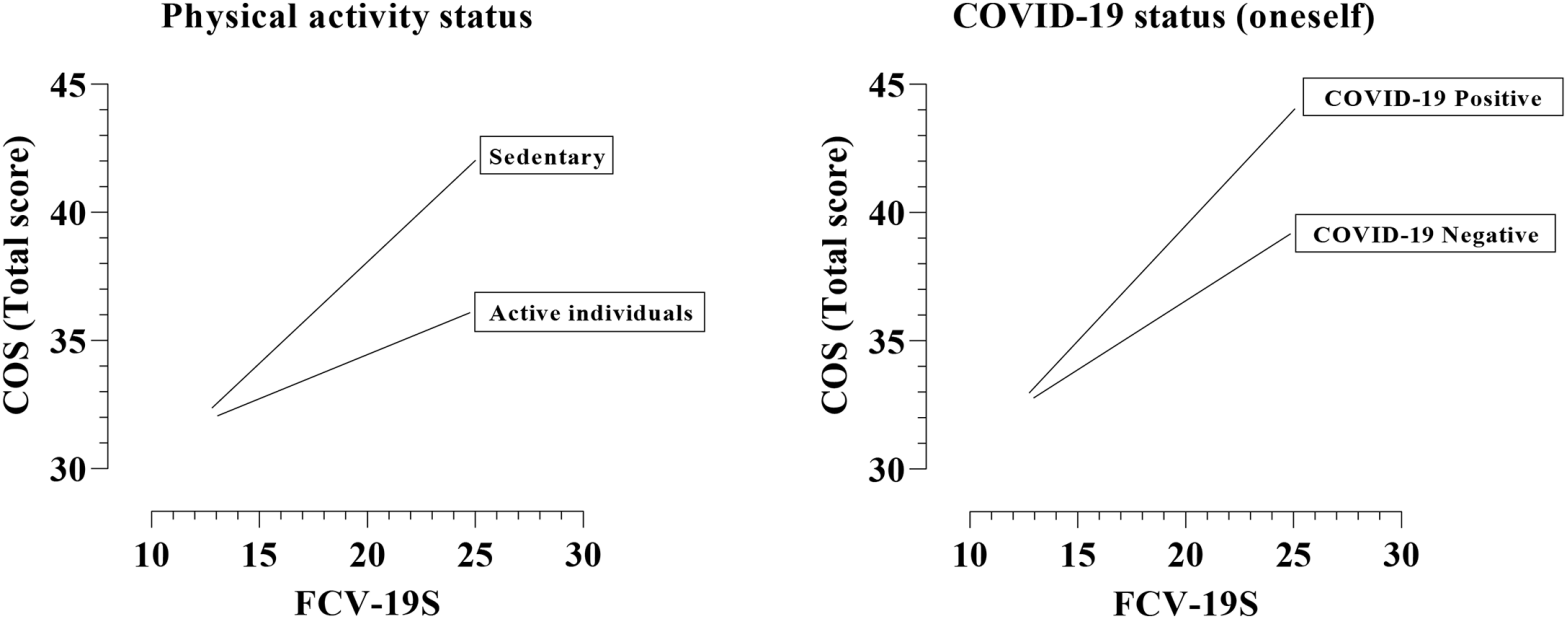
Moderating effect of physical activity and COVID-19 status in relation to FCV-19S and COS (slope plots). COS = Coping with Outbreak Scale; FCV-19S = Fear of COVID-19 Scale.

## Results

The descriptive characteristics of participants are summarized in Table 1. The findings of the study revealed a positive correlation between the total scores of the FCV-19S and COS (ρ = 0.451, p < 0.001). Furthermore, FCV-19S exhibited significant correlations with sub-dimensions, which cognitive (ρ = 0.259), transcendental (ρ = 0.320), behavioral (ρ = 0.239), and relational coping (ρ = 0.237) (p < 0.001 for all) (Fig 2).

**Table 1:** Descriptive characteristics of participants.

	N	Mean (SD) or %
**Characteristics**		
Age, years	1000	21.5 (2.5)
**Physical activity status**		
Recreationally active individual	247	24.7
Sedentary	753	75.3
**COVID-19 status of participants**		
COVID-19 positive	274	27.4
COVID-19 negative	726	72.6
**COVID-19 status of relatives**		
COVID-19 positive	490	49
COVID-19 negative	510	51
**COVID-19 status of close friends**		
COVID-19 positive	764	76.4
COVID-19 negative	236	23.6
**Relatives or close friends who died from COVID-19**		
Yes	536	53.6
No	464	46.4
**Emotional response to meeting with a post-COVID survivor**		
I get worried	314	31.4
I am comfortable	686	68.6
**COVID-19 vaccination status**		
Vaccinated	988	98.8
Unvaccinated	12	1.2
**Number of vaccine doses**		
Dose 1	26	2.6
Dose 2	962	96.2
**Disease severity in COVID-19 patients**		
Mildly	97	9.7
Moderate	134	13.4
Severe	43	4.3
**Measurements**		
FCV-19S		18.9 (7.0)
COS (Total score)		36.6 (9.7)
COS – Cognitive		7.6 (3.7)
COS – Transcendental		11.0 (4.9)
COS – Behavioral		8.4 (3.6)
COS – Relational		9.6 (5.0)

SD = Standard deviation; COS = Coping with Outbreak Scale; FCV-19S = Fear of COVID-19 Scale

To test hypothesis 2, we interrogated the potential moderating roles of all descriptive characteristics in the relationship between the fear of COVID-19 and related coping strategies. Table 2 shows that physical activity and COVID-19 status had a moderating effect on participants’ coping strategies used in response to COVID-19 fear (p < 0.050). The results indicated that the physical activity status significantly moderated the relationship between the FCV-19S and COS (*B* = −6.97, p < 0.001). Accordingly, the simple slope analysis revealed a significant difference in the effect between sedentary (simple slope = 0.79, t = 17.877, p < 0.050) and recreationally active individuals (simple slope = 0.28, t = 3.79, p < 0.001), highlighting the varying impact of physical activity status on the COVID-19 fear and the coping strategies (Fig 3). Moreover, the PROCESS Macro analysis revealed a significant interaction effect between the fear of COVID-19 and physical activity status (FCV-19 x MV_1_) on the coping strategies employed with a regression coefficient of *B* = 0.51, indicating statistical significance (p < 0.001). The MV_1_ predictor in the model explained 26% of the variance in the COS variable (Table 2).

**Table 2:** Moderating effects of physical activity and COVID-19 status on the relationship between FCV-19S and COS.

Variable entered	COS			
	*B*	SE	t	95% CI
FCV-19S	−0.23	0.15	−1.49	[-0.53 to 0.07]
Physical activity status (MV_1_)	−6.97	1.70**	−4.10	[-10.31 to -3.64]
FCV-19S x MV_1_	0.51	0.09**	5.91	[0.34 to 0.68]
MV_1_ – Model summary	*R*^*2*^ = 0.26, *ΔR*^*2*^ = 0.025, *F* = 34.984			
FCV-19S	1.31	0.14	9.29	[1.04 to 1.59]
COVID-19 status (oneself) (MV_2_)	4.98	1.66*	3.00	[1.72 to 8.24]
FCV-19S x MV_2_	−0.39	0.08**	−4.77	[-0.55 to -0.23]
MV_2_ – Model summary	*R*^*2*^ = 0.26, *ΔR*^*2*^ = 0.017, *F* = 22.780			

*B* = Unstandardized regression coefficients; COS = Coping with Outbreak Scale; F  =  F-test; SE =  standard error; t  =  t-test score; 95% CI = confidence interval; MV: Moderate variable; *p  < 0.050; **p  < 0.001

Our study findings also showed that participants’ COVID-19 status had a moderating effect on the relationship between fear and coping strategies (*B* = 4.98, p < 0.050). The simple slope analysis indicated a significant difference in the impact between COVID-19 positive (simple slope = 0.92, t = 13.856, p < 0.001) and negative individuals (simple slope = 0.53, t = 11.583, p < 0.001) (Fig 3). Additionally, the interaction effect between fear of COVID-19 and COVID-19 status (FCV-19 x MV_2_) had a significant influence on coping strategies (*B* = −0.39, p < 0.001). The MV_2_ predictor in the model accounted for 26% of the variance in the COS variable (Table 2).

## Discussion

The primary aim of this study was to investigate the relationship between fear of COVID-19 and coping strategies among female university students and to determine whether selected individual characteristics moderated this relationship. This study provides novel findings demonstrating the relationship between fear of COVID-19 and related coping strategies in female university students, and highlighting the factors moderating this relationship. Our results indicated a positive association between fear of COVID-19 and coping strategies, which is in alignment with the first hypothesis. Furthermore, the study reveals significant associations between fear of COVID-19 and sub-dimensions of coping, including cognitive, transcendental, behavioral and relational coping. These results suggest that individuals may develop both more and various coping strategies as fear levels increase.

The intricate relationship between fear of COVID-19 and coping strategies can be interpreted by the broader paradigm of stress and coping theory proposed by Lazarus and Folkman [14]. Stress is perceived as a relationship between the person and their environment and is viewed by the person as taxing or exceeding his/her resources and putting his/her well-being at risk. Coping encompasses the dynamic cognitive and behavioral efforts used to manage specific external and/or internal demands [14]. Numerous studies have indicated that the lethal danger posed by COVID-19 to human health has caused fear, a potential environmental stressor among people [5,6]. This stressor, known as the fear of COVID-19, quickly became a significant psychological concern that profoundly affects people’s mental health leading to issues like depression, anxiety, and stress [10,29,30]. Furthermore, a recent study supported a notable prevalence of fear-related mood states, including emotional disturbance, irritability, and anger, among individuals in the post-pandemic phase [31]. As an emotion, fear can trigger a variety of psychological responses and coping strategies to deal with dangerous or threatening situations, activating the instinctive and physical behaviors of the individual [11,29]. Our findings suggest a positive correlation between the fear of COVID-19 and coping strategies among female university students during the post-emergency phase. Perceiving COVID-19 infection as a major threat in our female cohort might have induced a fear response, prompting them to develop more effective coping strategies. We also reported that the transcendence sub-dimension was favoured as the most used coping strategies in the relationship between COVID-19 and pandemic coping strategies in this cohort.

The findings regarding the moderating roles of physical activity status and previous COVID-19 infection experience emphasize the importance of individual differences in coping with the COVID-19-related concerns. Our findings were also in line with the second hypothesis, we observed that physical activity status plays a moderating role in the relationship between fear of COVID-19 and coping with the outbreak. In particular, the moderating effect of individuals’ physical activity status on fear of COVID-19 and coping levels is more pronounced in sedentary individuals (vs. recreationally active individuals). Previous studies have reported that regular exercise positively supports individuals’ mental health components such as psychological resilience, well-being, stress management and self-efficacy perceptions [32–34]. However, regular physical activity has also been demonstrated to considerably reduce depression and anxiety levels and to have improving effects on post-traumatic symptoms [35–37]. Senışık and colleagues [37] stated that athletes survived the pandemic with less mental strain compared to sedentary individuals and that regular exercise habits play an important role in reducing psychological distress during challenging times [38]. Likewise, several studies have shown that regular physical activity before or during the COVID-19 pandemic is associated with better mental health outcomes such as psychological flexibility and well-being in young people [39–41]. A few studies suggest that psychological flexibility and psychological well-being related to adaptive coping may play a moderate role in this relationship [42–44]. Psychological flexibility refers to an individual’s ability to adapt and respond effectively to challenging or stressful situations [44], which may be an important factor in understanding and predicting how individuals overcome the challenges posed by the COVID-19-related concerns. The steeper positive slope observed among sedentary individuals suggests that their coping responses were more strongly contingent on increasing fear of COVID-19. More specifically, sedentary individuals may have been more likely to activate coping strategies when fear became more salient. By contrast, the flatter but still significant slope among recreationally active individuals may indicate that their coping responses were less dependent on elevated fear levels. This pattern may reflect a more stable baseline repertoire of adaptive coping resources among active individuals, potentially supported by the beneficial effects of regular physical activity on psychological resilience, well-being, stress regulation, and self-efficacy. Therefore, physical activity may not simply reduce fear or coping needs; rather, it may alter the extent to which coping responses are driven by fear. However, it is also important to emphasize that multiple factors, including personal experiences, individual differences, and pre-existing health conditions may also contribute to this relationship. Nevertheless, further research is needed to identify factors that moderate the relationship between fear of COVID-19 and related coping responses.

On the other hand, we reported that individuals’ COVID-19 status (positive or negative) may have a moderating role in the relationship between fear of COVID-19 and coping levels with the pandemic. In particular, the moderate effect of individuals’ infection status on COVID-19 fear and coping levels is more pronounced in positive individuals (vs. negatives). Positive cases may experience high fear and anxiety due to their direct experience with the virus, which can lead to stress, anxiety and affect their general well-being. Several studies have reported more severe symptoms of stress, anxiety and depression in people who reported a positive COVID-19 test, including those who did not require hospitalisation [45,46]. Likewise, Ewid et al. found that stress, anxiety, and depression levels were significantly higher in students who had close contact with COVID-19 cases and whose lives were affected by COVID-19 compared to their peers [47]. Our findings align with the notion that an individual’s infection status can profoundly impact their psychological state and, consequently, their coping strategies. People who directly experience the physical and psychological damages of the virus may be more inclined to utilise active coping strategies. González-Castro et al. argued that an individual’s perceived vulnerability to a disease, combined with their perception of the disease’s severity, can influence their motivation to adopt health protective behaviors [48]. Thus, COVID-19 positive individuals might perceive the disease as more severe and sensitive than those who are negative, which may elevate their level of fear, causing them to develop various or more coping strategies. In addition, Koçak et al. noted that having PCR-positive friends or family had a moderate effect on fear of COVID-19, which causes depression, anxiety, and stress in people [30]. This suggests that the stigma associated with a positive diagnosis may increase fear and shape coping behaviors even among those who test negative. Nevertheless, it should also be acknowledged that more than half of the participants reported the loss of a relative or close friend due to COVID-19. Such bereavement-related experiences may have influenced fear perceptions and coping responses independently of participants’ own infection history and therefore represent a potential source of unmeasured psychological variability.

### Strength and limitations

One strength of our study is its addition to the literature on COVID-19 related fear and coping, with a specific focus on a young, exclusively female demographic. This research further reinforces the limited literature that investigates both direct and indirect influences in identifying key modulating factors between fear and coping strategies during the post-emergency phase. Nevertheless, the findings of this study should be considered in light of some limitations. Most importantly, a cross-sectional design was used for this study, thus causal interpretations about the relationships between variables are quite limited. Another potential limitation was the use of self-report measures. Participants might have under-or-over-reported their experiences which are potentially susceptible to scientifically desirable outcomes. To enhance the reliability of self-report responses, participants were assured of their anonymity and confidentiality, aiming to increase the honesty and objectivity of their feedback. Finally, the use of convenience sampling limits the extent to which these findings can be generalized beyond the specific cohort of female university students who participated in the study. However, future studies using randomized or stratified sampling methods could be valuable in determining whether these results are potentially applicable to other populations.

### Future directions

The findings of this study provide important insights for the management of future public health crises, particularly in light of the experiences gained from the COVID-19 pandemic. It emphasizes the critical importance of robust surveillance systems and early intervention strategies to mitigate the long- or short-term psychological effects that may arise from the infection disproportionately affecting potentially susceptible groups [49,50]. The results contribute to a better understanding of the factors influencing coping strategies during global health crises, particularly when systemic weaknesses—such as inequalities in access to healthcare and vaccine distribution—become apparent. In the future, incorporating key factors that influence coping strategies—such as physical activity—into global health preparedness plans could be critical for strengthening resilience against future pandemics.

## Conclusion

In this study, we provided new insights into the relationship between fear of COVID-19 and coping strategies among female university students, highlighting the role of moderating factors in this relationship. Our results emphasize a positive correlation between fear of COVID-19 and COS strategies, suggesting that individuals may adopt diverse coping responses based on their level of fear. Furthermore, we reported the crucial role of physical activity status and COVID-19 positive or negative experiences as key moderators in relationship between the fear and coping during the post-emergency phase. These findings suggest that individual characteristics—like sedentary lifestyle habits and firsthand experiences with the infection—may intensify fear responses, thereby shaping coping strategies. Consequently, this model can guide professionals’ interventions to develop and improve coping dynamics among people in response to the new challenges posed by stressful events or public health crises such as COVID-19 infection in their psycho-social context.
