# Supplementary material for: Factors associated with fear and coping strategies during pandemic in female university students: The moderator role of physical activity and infection status
Source: PLoS One. 2026 Aug 11;21(8):e0355544. doi: 10.1371/journal.pone.0355544 (PMC13460565; doi:10.1371/journal.pone.0355544)
Supplement: Supplementary file 2 [file pone.0355544.s003.docx]

**Point-by-point response addressing queries from the reviewer**

Ref.: ONE-D-26-19080R2

Factors associated with fear and coping strategies during pandemic in female university students: The moderator role of physical activity and infection status

**Reviewer 1 & 2**

We sincerely thank Reviewers 1 and 2 for their careful re-evaluation of our revised manuscript and for their positive feedback. We greatly appreciate their recognition of the revisions made in response to the initial comments. Their constructive suggestions have significantly improved the quality and clarity of our manuscript, and we are grateful for the time and effort they devoted to the review process.

Ref.: ONE-D-26-19080R1

Factors associated with fear and coping strategies during pandemic in female university students: The moderator role of physical activity and infection status

**Reviewer 1**

Thank you for reviewing our paper and providing constructive comments. We have done our best to address your queries in this version.

**Comment #1:**

It is recommended that the introduction conclude with a clear statement of the paper's purpose, a summary of its novel contributions, and a description of its position within the specialized scientific literature.

Response #1:

Thank you for this valuable suggestion. We have revised the final paragraph of the Introduction to more clearly state the study purpose, highlight its novel contributions, and position the study within the existing literature on fear and coping strategies (please see page 3, lines 70-81 in the marked version of the manuscript).

**Comment #2:**

The statistical software should be cited as follows: GraphPad Software. (2023). GraphPad Prism (Version 9) [Computer software]. San Diego, CA: GraphPad Software, Inc. Available at <https://www.graphpad.com>.

Response #2:

Thank you for the suggestion. The recommended citation for GraphPad Prism has been added to the statistical analysis section (please see page 7, lines 167-168 in the marked version of the manuscript).

**Comment #3:**

The discussion section should begin by restating the study's purpose and explicitly indicating whether this objective was achieved.

Response #3:

Thank you for this suggestion. The opening paragraph of the Discussion has been revised to clearly restate the study purpose (please see page 10, lines 212-215 in the marked version of the manuscript).

**Comment #4:**

Additionally, a subchapter titled "Study strengths, limitations, future directions, and clinical implications" should be included, or these topics should be addressed in separate subsections within the discussion section.

Response #4:

Thank you for this recommendation. We have reorganized the final section of the Discussion and added a dedicated subsection entitled “Study strengths, limitations, and future directions” to improve clarity and align with journal recommendations (please see pages 13-14, lines 297-321 in the marked version of the manuscript).

________________________________________________________________________________

**Reviewer 2**

Thank you for reviewing our paper and providing constructive comments. We have done our best to address your queries in this version.

**Comment #1:**

**a)** Critical Contradiction in Sample Description

There is a fundamental contradiction between the text in the Participants and Procedure section and the data presented in Table 1:

- Text statement: "All participants included in the study were laboratory-confirmed. Our cohort reported being vaccinated before infection." This phrasing strongly implies that 100% of the sample contracted COVID-19.

-Table 1 data: Indicates that only 27.4% (N=274) were COVID-19 positive, while 72.6% (N=726) were COVID-19 negative.

- Reviewer Action Item: The authors must explicitly clarify what "laboratory-confirmed" refers to. If it means verified PCR status (regardless of whether positive or negative), the wording must be heavily revised to avoid misleading readers into thinking it was an entirely post-infection cohort.

Response #1a:

Thank you for highlighting this ambiguity. We agree that the original wording could be misinterpreted as indicating that all participants had previously contracted COVID-19. Our intention was to specify that only participants classified as COVID-19 positive reported a laboratory-confirmed diagnosis. To improve clarity, we revised the manuscript to indicate that COVID-19 infection status was based on laboratory-confirmed diagnoses reported through the official Turkish Ministry of Health electronic health record system (e-Nabız), which is directly accessible to individuals (please see page 4, lines 95-98 in the marked version of the manuscript).

**b)** Timeline Contextualization (2023 vs. Active Pandemic)

The data collection period is stated as July–December 2023. By late 2023, the World Health Organization (WHO) had declared an end to COVID-19 as a global health emergency, and daily public restrictions had largely abated.

- The manuscript regularly frames the study as capturing experiences "during the outbreak" or addressing an active crisis context.

- Reviewer Action Item: The authors need to contextualize their discussion. Are participants recalling past trauma, or are they reacting to the tail-end landscape of 2023? The use of the word "outbreak" should be adjusted to accurately match the late-2023 epidemiological landscape.

Response #1b:

Thank you for this important observation. We agree that the original wording may not have adequately reflected the epidemiological context in which the data were collected. Although the acute phase of the pandemic had largely subsided by late 2023, COVID-19-related concerns, previous infection experiences, and associated psychological responses remained present among university students. Therefore, the present study reflects participants’ contemporaneous perceptions and coping responses during the post-emergency phase of the COVID-19 pandemic. To clarify this point, we revised the title, Abstract, Introduction, Participants and Procedure, Measures, Discussion, and Conclusion sections. We also replaced or qualified terms such as “during the outbreak” and “during the pandemic” with wording that more accurately reflects the post-emergency phase of COVID-19. Furthermore, we have added a detail to the ‘Methods’ section stating that the responses relate to the current situation regarding COVID-19 (please see page 5, lines 123-124 in the marked version of the manuscript).

**c)** Convoluted Moderation Logic in Discussion

- The interpretation of the physical activity moderation results requires deeper nuance: The simple slope analysis reveals that the positive relationship between fear (FCV-19S) and coping (COS) is significantly steeper for sedentary individuals (slope = 0.79) than for recreationally active individuals (slope = 0.28).

- The authors argue that active individuals have better mental health frameworks, which "contributed to our cohort experiencing less fear... and thus producing fewer coping strategies.

- "Reviewer Action Item: This logic is counterintuitive. Active individuals might actually possess a baseline level of adaptive coping mechanisms that operate independently of high fear states, meaning their coping behaviors do not rely heavily on being afraid. Conversely, sedentary individuals may only deploy coping mechanisms when fear reaches acute thresholds. The authors should revise this portion of the discussion to improve clarity and avoid contradictory statements.

Response #1c:

Thank you for this insightful comment. We have revised the Discussion to avoid implying that recreationally active individuals simply experienced less fear and therefore used fewer coping strategies. Instead, we now interpret the flatter slope among recreationally active individuals as suggesting that their coping responses may be less dependent on elevated fear levels, potentially reflecting more stable or pre-existing adaptive coping resources. Conversely, the steeper slope among sedentary individuals may indicate that coping responses become more strongly activated as fear increases (please see page 12, lines 259-271 in the marked version of the manuscript).

**Comment #2:**

**a)** Typographical and Nomenclature Consistency

- Variable Names: In the text describing the PROCESS Macro results for MV1 and MV2, the authors refer to the dependent variable as "the COP variable". However, throughout the rest of the paper, the scale is abbreviated as COS (Coping with Outbreak Scale). This must be standardized to "COS" across the entire manuscript.

- Statistical Abbreviations: Ensure that standard symbols are formatted uniformly (e.g., matching the use of unstandardized regression coefficients $B$ versus standardized betas, or standardizing alpha levels across sections).

Response #2a:

Thank you for identifying this inconsistency. We have corrected all references to ‘‘COP’’ and replaced them with the appropriate abbreviation, ‘‘COS,’’ throughout the manuscript to ensure consistency. In addition, we reviewed the reporting of statistical notation and harmonized the presentation of statistical symbols, regression coefficients (*B*), and significance levels across the text, tables, and figure legends (please see page 9, lines 184-206 in the marked version of the manuscript).

**b)** Clarification on "Relatives or Close Friends Who Died"

- Table 1 shows that 53.6% (N=536) of the participants reported having a relative or close friend who died from COVID-19. This is an extraordinarily high proportion for a single convenience sample.

- Reviewer Action Item: The authors should offer brief context regarding regional or local mortality impacts near the University of Ondokuz Mayıs during peak waves to ground this figure, as this variable likely represents a massive confounding psychological stressor that could cloud the specific impact of personal infection status (MV2).

Response #2b:

Thank you for this observation. We agree that the proportion of participants reporting the loss of a relative or close friend due to COVID-19 appears substantial. However, the survey item combined both relatives and close friends, thereby capturing losses occurring within a broader social network rather than only within the immediate family. Given the widespread impact of COVID-19 across Türkiye during the pandemic years, such experiences were relatively common among young adults. To provide additional context and avoid overinterpretation, we have clarified this point in the manuscript and acknowledged that bereavement-related experiences may have contributed to participants’ psychological responses during the study period (please see page 13, lines 292-296 in the marked version of the manuscript).

**Comment #3:**

- Summary Evaluation: This manuscript addresses an important demographic (young female university students) and evaluates useful protective behavioral variables (physical activity). The sample size (N=1000) provides excellent statistical power for the moderation models applied. However, the manuscript cannot be accepted in its current state due to the blatant text-to-table contradiction regarding participant infection status and the lack of historical/epidemiological anchoring for a study conducted in late 2023. Resolving these narrative and data alignment problems is essential prior to publication.

Response #3:

Thank you for your constructive assessment of our manuscript. In response to these concerns, we thoroughly revised the manuscript to resolve the inconsistency regarding participant infection status and to better contextualize the study within the late-2023 post-emergency phase of COVID-19. We believe that these revisions have substantially improved the clarity, consistency, and interpretability of the manuscript.
