# Supplementary material for: Factors associated with fear and coping strategies during pandemic in female university students: The moderator role of physical activity and infection status
Source: PLoS One. 2026 Aug 11;21(8):e0355544. doi: 10.1371/journal.pone.0355544 (PMC13460565; doi:10.1371/journal.pone.0355544)
Supplement: Peer review history

# Peer review: Decision Letter 0

**DOI:** 10.1371/journal.pone.0355544.r001

**Authors:** Wanli Zang

21 Jun 2026

PONE-D-26-19080

Factors associated with fear and coping strategies during pandemic in female university students: The moderator role of physical activity and infection status

PLOS One

Dear Dr. ERMİŞ,

Thank you for submitting your manuscript to PLOS ONE. After careful consideration, we feel that it has merit but does not fully meet PLOS ONE’s publication criteria as it currently stands. Therefore, we invite you to submit a revised version of the manuscript that addresses the points raised during the review process.

Please submit your revised manuscript by Aug 05 2026 11:59PM. If you will need more time than this to complete your revisions, please reply to this message or contact the journal office at plosone@plos.org. When you're ready to submit your revision, log on to https://www.editorialmanager.com/pone/ and select the 'Submissions Needing Revision' folder to locate your manuscript file.

Please include the following items when submitting your revised manuscript:

- A letter that responds to each point raised by the academic editor and reviewer(s). You should upload this letter as a separate file labeled 'Response to Reviewers'.
- A marked-up copy of your manuscript that highlights changes made to the original version. You should upload this as a separate file labeled 'Revised Manuscript with Track Changes'.
- An unmarked version of your revised paper without tracked changes. You should upload this as a separate file labeled 'Manuscript'.

If you would like to make changes to your financial disclosure, please include your updated statement in your cover letter. Guidelines for resubmitting your figure files are available below the reviewer comments at the end of this letter.

If applicable, we recommend that you deposit your laboratory protocols in protocols.io to enhance the reproducibility of your results. Protocols.io assigns your protocol its own identifier (DOI) so that it can be cited independently in the future. For instructions see: https://journals.plos.org/plosone/s/submission-guidelines#loc-laboratory-protocols. Additionally, PLOS ONE offers an option for publishing peer-reviewed Lab Protocol articles, which describe protocols hosted on protocols.io. Read more information on sharing protocols at https://plos.org/protocols?utm_medium=editorial-email&utm_source=authorletters&utm_campaign=protocols.

As the corresponding author, your ORCID iD is verified in the submission system and will appear in the published article. PLOS supports the use of ORCID, and we encourage all coauthors to register for an ORCID iD and use it as well. Please encourage your coauthors to verify their ORCID iD within the submission system before final acceptance, as unverified ORCID iDs will not appear in the published article. *Only*  the individual author can complete the verification step; PLOS staff *cannot*  verify ORCID iDs on behalf of authors.

We look forward to receiving your revised manuscript.

Kind regards,

Guest Editor

PLOS One

Journal Requirements:

When submitting your revision, we need you to address these additional requirements.

1. Please ensure that your manuscript meets PLOS ONE's style requirements, including those for file naming. The PLOS ONE style templates can be found at

https://journals.plos.org/plosone/s/file?id=wjVg/PLOSOne_formatting_sample_main_body.pdf and

https://journals.plos.org/plosone/s/file?id=ba62/PLOSOne_formatting_sample_title_authors_affiliations.pdf.

2. In the online submission form you indicate that your data is not available for proprietary reasons and have provided a contact point for accessing this data. Please note that your current contact point is a co-author on this manuscript. According to our Data Policy, the contact point must not be an author on the manuscript and must be an institutional contact, ideally not an individual. Please revise your data statement to a non-author institutional point of contact, such as a data access or ethics committee, and send this to us via return email. Please also include contact information for the third party organization, and please include the full citation of where the data can be found.

3. If the reviewer comments include a recommendation to cite specific previously published works, please review and evaluate these publications to determine whether they are relevant and should be cited. There is no requirement to cite these works unless the editor has indicated otherwise.

[Note: HTML markup is below. Please do not edit.]

Reviewers' comments:

Reviewer's Responses to Questions

**Comments to the Author**

1. Is the manuscript technically sound, and do the data support the conclusions?

The manuscript must describe a technically sound piece of scientific research with data that supports the conclusions. Experiments must have been conducted rigorously, with appropriate controls, replication, and sample sizes. The conclusions must be drawn appropriately based on the data presented.

Reviewer #1: No

Reviewer #2: Partly

**********

2. Has the statistical analysis been performed appropriately and rigorously?

Reviewer #1: Yes

Reviewer #2: Yes

**********

3. Have the authors made all data underlying the findings in their manuscript fully available?

The PLOS Data policy requires authors to make all data underlying the findings described in their manuscript fully available without restriction, with rare exception (please refer to the Data Availability Statement in the manuscript PDF file). The data should be provided as part of the manuscript or its supporting information, or deposited to a public repository. For example, in addition to summary statistics, the data points behind means, medians and variance measures should be available. If there are restrictions on publicly sharing data—e.g. participant privacy or use of data from a third party—those must be specified.

Reviewer #1: Yes

Reviewer #2: Yes

**********

4. Is the manuscript presented in an intelligible fashion and written in standard English?

PLOS ONE does not copyedit accepted manuscripts, so the language in submitted articles must be clear, correct, and unambiguous. Any typographical or grammatical errors should be corrected at revision, so please note any specific errors here.

Reviewer #1: Yes

Reviewer #2: Yes

**********

5. Review Comments to the Author

Please use the space provided to explain your answers to the questions above. You may also include additional comments for the author, including concerns about dual publication, research ethics, or publication ethics. (Please upload your review as an attachment if it exceeds 20,000 characters)

Reviewer #1: • It is recommended that the introduction conclude with a clear statement of the paper's purpose, a summary of its novel contributions, and a description of its position within the specialized scientific literature.

• The statistical software should be cited as follows: GraphPad Software. (2023). GraphPad Prism (Version 9) [Computer software]. San Diego, CA: GraphPad Software, Inc. Available at https://www.graphpad.com.

• The discussion section should begin by restating the study's purpose and explicitly indicating whether this objective was achieved.

• Additionally, a subchapter titled "Study strengths, limitations, future directions, and clinical implications" should be included, or these topics should be addressed in separate subsections within the discussion section.

Reviewer #2: Manuscript ID: PONE-D-26-19080

Manuscript Title: "Factors associated with fear and coping strategies during pandemic in female university students: The moderator role of physical activity and infection status"

1. Major Revisions & Methodological Concerns

a) Critical Contradiction in Sample Description

There is a fundamental contradiction between the text in the Participants and Procedure section and the data presented in Table 1:

- Text statement: "All participants included in the study were laboratory-confirmed. Our cohort reported being vaccinated before infection." This phrasing strongly implies that 100% of the sample contracted COVID-19.

--Table 1 data: Indicates that only 27.4% (N=274) were COVID-19 positive, while 72.6% (N=726) were COVID-19 negative.

- Reviewer Action Item: The authors must explicitly clarify what "laboratory-confirmed" refers to. If it means verified PCR status (regardless of whether positive or negative), the wording must be heavily revised to avoid misleading readers into thinking it was an entirely post-infection cohort.

b) Timeline Contextualization (2023 vs. Active Pandemic)

The data collection period is stated as July–December 2023. By late 2023, the World Health Organization (WHO) had declared an end to COVID-19 as a global health emergency, and daily public restrictions had largely abated.

- The manuscript regularly frames the study as capturing experiences "during the outbreak" or addressing an active crisis context.

- Reviewer Action Item: The authors need to contextualize their discussion. Are participants recalling past trauma, or are they reacting to the tail-end landscape of 2023? The use of the word "outbreak" should be adjusted to accurately match the late-2023 epidemiological landscape.

c) Convoluted Moderation Logic in Discussion

- The interpretation of the physical activity moderation results requires deeper nuance:The simple slope analysis reveals that the positive relationship between fear (FCV-19S) and coping (COS) is significantly steeper for sedentary individuals (slope = 0.79) than for recreationally active individuals (slope = 0.28).

- The authors argue that active individuals have better mental health frameworks, which "contributed to our cohort experiencing less fear... and thus producing fewer coping strategies.

- "Reviewer Action Item: This logic is counterintuitive. Active individuals might actually possess a baseline level of adaptive coping mechanisms that operate independently of high fear states, meaning their coping behaviors do not rely heavily on being afraid. Conversely, sedentary individuals may only deploy coping mechanisms when fear reaches acute thresholds. The authors should revise this portion of the discussion to improve clarity and avoid contradictory statements.

2. Minor Revisions & Data Reporting Adjustments

a) Typographical and Nomenclature Consistency

- Variable Names: In the text describing the PROCESS Macro results for MV1 and MV2, the authors refer to the dependent variable as "the COP variable". However, throughout the rest of the paper, the scale is abbreviated as COS (Coping with Outbreak Scale). This must be standardized to "COS" across the entire manuscript.

- Statistical Abbreviations: Ensure that standard symbols are formatted uniformly (e.g., matching the use of unstandardized regression coefficients $B$ versus standardized betas, or standardizing alpha levels across sections).

b) Clarification on "Relatives or Close Friends Who Died"

- Table 1 shows that 53.6% (N=536) of the participants reported having a relative or close friend who died from COVID-19. This is an extraordinarily high proportion for a single convenience sample.

- Reviewer Action Item: The authors should offer brief context regarding regional or local mortality impacts near the University of Ondokuz Mayıs during peak waves to ground this figure, as this variable likely represents a massive confounding psychological stressor that could cloud the specific impact of personal infection status (MV2).

3. Reviewer Conclusion & Recommendation

- Recommendation: Major Revision

- Summary Evaluation:This manuscript addresses an important demographic (young female university students) and evaluates useful protective behavioral variables (physical activity). The sample size (N=1000) provides excellent statistical power for the moderation models applied. However, the manuscript cannot be accepted in its current state due to the blatant text-to-table contradiction regarding participant infection status and the lack of historical/epidemiological anchoring for a study conducted in late 2023. Resolving these narrative and data alignment problems is essential prior to publication.

**********

6. PLOS authors have the option to publish the peer review history of their article (what does this mean?). If published, this will include your full peer review and any attached files.

If you choose “no”, your identity will remain anonymous but your review may still be made public.

**Do you want your identity to be public for this peer review?**  For information about this choice, including consent withdrawal, please see our Privacy Policy.

Reviewer #1: No

Reviewer #2: **Yes:** Dr Rimah Melati Ab Ghani

**********

[NOTE: If reviewer comments were submitted as an attachment file, they will be attached to this email and accessible via the submission site. Please log into your account, locate the manuscript record, and check for the action link "View Attachments". If this link does not appear, there are no attachment files.]

To ensure your figures meet our technical requirements, please review our figure guidelines: https://journals.plos.org/plosone/s/figures

You may also use PLOS’s free figure tool, NAAS, to help you prepare publication quality figures: https://journals.plos.org/plosone/s/figures#loc-tools-for-figure-preparation.

NAAS will assess whether your figures meet our technical requirements by comparing each figure against our figure specifications.

# Author comment: Author response to Decision Letter 1

**DOI:** 10.1371/journal.pone.0355544.r002

23 Jun 2026

Point-by-point response addressing queries from the reviewer

Ref.: ONE-D-26-19080

Factors associated with fear and coping strategies during pandemic in female university students: The moderator role of physical activity and infection status

Reviewer 1

Thank you for reviewing our paper and providing constructive comments. We have done our best to address your queries in this version.

Comment #1:

It is recommended that the introduction conclude with a clear statement of the paper's purpose, a summary of its novel contributions, and a description of its position within the specialized scientific literature.

Response #1:

Thank you for this valuable suggestion. We have revised the final paragraph of the Introduction to more clearly state the study purpose, highlight its novel contributions, and position the study within the existing literature on fear and coping strategies (please see page 3, lines 70-81 in the marked version of the manuscript).

Comment #2:

The statistical software should be cited as follows: GraphPad Software. (2023). GraphPad Prism (Version 9) [Computer software]. San Diego, CA: GraphPad Software, Inc. Available at https://www.graphpad.com.

Response #2:

Thank you for the suggestion. The recommended citation for GraphPad Prism has been added to the statistical analysis section (please see page 7, lines 167-168 in the marked version of the manuscript).

Comment #3:

The discussion section should begin by restating the study's purpose and explicitly indicating whether this objective was achieved.

Response #3:

Thank you for this suggestion. The opening paragraph of the Discussion has been revised to clearly restate the study purpose (please see page 10, lines 212-215 in the marked version of the manuscript).

Comment #4:

Additionally, a subchapter titled "Study strengths, limitations, future directions, and clinical implications" should be included, or these topics should be addressed in separate subsections within the discussion section.

Response #4:

Thank you for this recommendation. We have reorganized the final section of the Discussion and added a dedicated subsection entitled “Study strengths, limitations, and future directions” to improve clarity and align with journal recommendations (please see pages 13-14, lines 297-321 in the marked version of the manuscript).

________________________________________________________________________________

Reviewer 2

Thank you for reviewing our paper and providing constructive comments. We have done our best to address your queries in this version.

Comment #1:

a) Critical Contradiction in Sample Description

There is a fundamental contradiction between the text in the Participants and Procedure section and the data presented in Table 1:

- Text statement: "All participants included in the study were laboratory-confirmed. Our cohort reported being vaccinated before infection." This phrasing strongly implies that 100% of the sample contracted COVID-19.

-Table 1 data: Indicates that only 27.4% (N=274) were COVID-19 positive, while 72.6% (N=726) were COVID-19 negative.

- Reviewer Action Item: The authors must explicitly clarify what "laboratory-confirmed" refers to. If it means verified PCR status (regardless of whether positive or negative), the wording must be heavily revised to avoid misleading readers into thinking it was an entirely post-infection cohort.

Response #1a:

Thank you for highlighting this ambiguity. We agree that the original wording could be misinterpreted as indicating that all participants had previously contracted COVID-19. Our intention was to specify that only participants classified as COVID-19 positive reported a laboratory-confirmed diagnosis. To improve clarity, we revised the manuscript to indicate that COVID-19 infection status was based on laboratory-confirmed diagnoses reported through the official Turkish Ministry of Health electronic health record system (e-Nabız), which is directly accessible to individuals (please see page 4, lines 95-98 in the marked version of the manuscript).

b) Timeline Contextualization (2023 vs. Active Pandemic)

The data collection period is stated as July–December 2023. By late 2023, the World Health Organization (WHO) had declared an end to COVID-19 as a global health emergency, and daily public restrictions had largely abated.

- The manuscript regularly frames the study as capturing experiences "during the outbreak" or addressing an active crisis context.

- Reviewer Action Item: The authors need to contextualize their discussion. Are participants recalling past trauma, or are they reacting to the tail-end landscape of 2023? The use of the word "outbreak" should be adjusted to accurately match the late-2023 epidemiological landscape.

Response #1b:

Thank you for this important observation. We agree that the original wording may not have adequately reflected the epidemiological context in which the data were collected. Although the acute phase of the pandemic had largely subsided by late 2023, COVID-19-related concerns, previous infection experiences, and associated psychological responses remained present among university students. Therefore, the present study reflects participants’ contemporaneous perceptions and coping responses during the post-emergency phase of the COVID-19 pandemic. To clarify this point, we revised the title, Abstract, Introduction, Participants and Procedure, Measures, Discussion, and Conclusion sections. We also replaced or qualified terms such as “during the outbreak” and “during the pandemic” with wording that more accurately reflects the post-emergency phase of COVID-19. Furthermore, we have added a detail to the ‘Methods’ section stating that the responses relate to the current situation regarding COVID-19 (please see page 5, lines 123-124 in the marked version of the manuscript).

c) Convoluted Moderation Logic in Discussion

- The interpretation of the physical activity moderation results requires deeper nuance: The simple slope analysis reveals that the positive relationship between fear (FCV-19S) and coping (COS) is significantly steeper for sedentary individuals (slope = 0.79) than for recreationally active individuals (slope = 0.28).

- The authors argue that active individuals have better mental health frameworks, which "contributed to our cohort experiencing less fear... and thus producing fewer coping strategies.

- "Reviewer Action Item: This logic is counterintuitive. Active individuals might actually possess a baseline level of adaptive coping mechanisms that operate independently of high fear states, meaning their coping behaviors do not rely heavily on being afraid. Conversely, sedentary individuals may only deploy coping mechanisms when fear reaches acute thresholds. The authors should revise this portion of the discussion to improve clarity and avoid contradictory statements.

Response #1c:

Thank you for this insightful comment. We have revised the Discussion to avoid implying that recreationally active individuals simply experienced less fear and therefore used fewer coping strategies. Instead, we now interpret the flatter slope among recreationally active individuals as suggesting that their coping responses may be less dependent on elevated fear levels, potentially reflecting more stable or pre-existing adaptive coping resources. Conversely, the steeper slope among sedentary individuals may indicate that coping responses become more strongly activated as fear increases (please see page 12, lines 259-271 in the marked version of the manuscript).

Comment #2:

a) Typographical and Nomenclature Consistency

- Variable Names: In the text describing the PROCESS Macro results for MV1 and MV2, the authors refer to the dependent variable as "the COP variable". However, throughout the rest of the paper, the scale is abbreviated as COS (Coping with Outbreak Scale). This must be standardized to "COS" across the entire manuscript.

- Statistical Abbreviations: Ensure that standard symbols are formatted uniformly (e.g., matching the use of unstandardized regression coefficients $B$ versus standardized betas, or standardizing alpha levels across sections).

Response #2a:

Thank you for identifying this inconsistency. We have corrected all references to ‘‘COP’’ and replaced them with the appropriate abbreviation, ‘‘COS,’’ throughout the manuscript to ensure consistency. In addition, we reviewed the reporting of statistical notation and harmonized the presentation of statistical symbols, regression coefficients (B), and significance levels across the text, tables, and figure legends (please see page 9, lines 184-206 in the marked version of the manuscript).

b) Clarification on "Relatives or Close Friends Who Died"

- Table 1 shows that 53.6% (N=536) of the participants reported having a relative or close friend who died from COVID-19. This is an extraordinarily high proportion for a single convenience sample.

- Reviewer Action Item: The authors should offer brief context regarding regional or local mortality impacts near the University of Ondokuz Mayıs during peak waves to ground this figure, as this variable likely represents a massive confounding psychological stressor that could cloud the specific impact of personal infection status (MV2).

Response #2b:

Thank you for this observation. We agree that the proportion of participants reporting the loss of a relative or close friend due to COVID-19 appears substantial. However, the survey item combined both relatives and close friends, thereby capturing losses occurring within a broader social network rather than only within the immediate family. Given the widespread impact of COVID-19 across Türkiye during the pandemic years, such experiences were relatively common among young adults. To provide additional context and avoid overinterpretation, we have clarified this point in the manuscript and acknowledged that bereavement-related experiences may have contributed to participants’ psychological responses during the study period (please see page 13, lines 292-296 in the marked version of the manuscript).

Comment #3:

- Summary Evaluation: This manuscript addresses an important demographic (young female university students) and evaluates useful protective behavioral variables (physical activity). The sample size (N=1000) provides excellent statistical power for the moderation models applied. However, the manuscript cannot be accepted in its current state due to the blatant text-to-table contradiction regarding participant infection status and the lack of historical/epidemiological anchoring for a study conducted in late 2023. Resolving these narrative and data alignment problems is essential prior to publication.

Response #3:

Thank you for your constructive assessment of our manuscript. In response to these concerns, we thoroughly revised the manuscript to resolve the inconsistency regarding participant infection status and to better contextualize the study within the late-2023 post-emergency phase of COVID-19. We believe that these revisions have substantially improved the clarity, consistency, and interpretability of the manuscript.

Attachment Submitted filename: Response to Reviewers.docx

# Peer review: Decision Letter 1

**DOI:** 10.1371/journal.pone.0355544.r003

**Authors:** Wanli Zang

17 Jul 2026

PONE-D-26-19080R1

Factors associated with fear and coping strategies during pandemic in female university students: The moderator role of physical activity and infection status

PLOS One

Dear Dr. ERMİŞ,

Thank you for submitting your manuscript to PLOS ONE. After careful consideration, we feel that it has merit but does not fully meet PLOS ONE’s publication criteria as it currently stands. Therefore, we invite you to submit a revised version of the manuscript that addresses the points raised during the review process.

Please submit your revised manuscript by Aug 31 2026 11:59PM. If you will need more time than this to complete your revisions, please reply to this message or contact the journal office at plosone@plos.org. When you're ready to submit your revision, log on to https://www.editorialmanager.com/pone/ and select the 'Submissions Needing Revision' folder to locate your manuscript file.

Please include the following items when submitting your revised manuscript:

- A letter that responds to each point raised by the academic editor and reviewer(s). You should upload this letter as a separate file labeled 'Response to Reviewers'.
- A marked-up copy of your manuscript that highlights changes made to the original version. You should upload this as a separate file labeled 'Revised Manuscript with Track Changes'.
- An unmarked version of your revised paper without tracked changes. You should upload this as a separate file labeled 'Manuscript'.

If you would like to make changes to your financial disclosure, please include your updated statement in your cover letter. Guidelines for resubmitting your figure files are available below the reviewer comments at the end of this letter.

If applicable, we recommend that you deposit your laboratory protocols in protocols.io to enhance the reproducibility of your results. Protocols.io assigns your protocol its own identifier (DOI) so that it can be cited independently in the future. For instructions see: https://journals.plos.org/plosone/s/submission-guidelines#loc-laboratory-protocols. Additionally, PLOS ONE offers an option for publishing peer-reviewed Lab Protocol articles, which describe protocols hosted on protocols.io. Read more information on sharing protocols at https://plos.org/protocols?utm_medium=editorial-email&utm_source=authorletters&utm_campaign=protocols.

As the corresponding author, your ORCID iD is verified in the submission system and will appear in the published article. PLOS supports the use of ORCID, and we encourage all coauthors to register for an ORCID iD and use it as well. Please encourage your coauthors to verify their ORCID iD within the submission system before final acceptance, as unverified ORCID iDs will not appear in the published article. *Only*  the individual author can complete the verification step; PLOS staff *cannot*  verify ORCID iDs on behalf of authors.

We look forward to receiving your revised manuscript.

Kind regards,

Wanli Zang, Ph.D.

Guest Editor

PLOS One

Journal Requirements:

1. If the reviewer comments include a recommendation to cite specific previously published works, please review and evaluate these publications to determine whether they are relevant and should be cited. There is no requirement to cite these works unless the editor has indicated otherwise.

2. Please review your reference list to ensure that it is complete and correct. If you have cited papers that have been retracted, please include the rationale for doing so in the manuscript text, or remove these references and replace them with relevant current references. Any changes to the reference list should be mentioned in the rebuttal letter that accompanies your revised manuscript. If you need to cite a retracted article, indicate the article’s retracted status in the References list and also include a citation and full reference for the retraction notice.

[Note: HTML markup is below. Please do not edit.]

Reviewers' comments:

Reviewer's Responses to Questions

**Comments to the Author**

1. If the authors have adequately addressed your comments raised in a previous round of review and you feel that this manuscript is now acceptable for publication, you may indicate that here to bypass the “Comments to the Author” section, enter your conflict of interest statement in the “Confidential to Editor” section, and submit your "Accept" recommendation.

Reviewer #1: All comments have been addressed

Reviewer #2: All comments have been addressed

**********

2. Is the manuscript technically sound, and do the data support the conclusions?

The manuscript must describe a technically sound piece of scientific research with data that supports the conclusions. Experiments must have been conducted rigorously, with appropriate controls, replication, and sample sizes. The conclusions must be drawn appropriately based on the data presented.

Reviewer #1: Yes

Reviewer #2: Yes

**********

3. Has the statistical analysis been performed appropriately and rigorously?

Reviewer #1: Yes

Reviewer #2: Yes

**********

4. Have the authors made all data underlying the findings in their manuscript fully available?

The PLOS Data policy requires authors to make all data underlying the findings described in their manuscript fully available without restriction, with rare exception (please refer to the Data Availability Statement in the manuscript PDF file). The data should be provided as part of the manuscript or its supporting information, or deposited to a public repository. For example, in addition to summary statistics, the data points behind means, medians and variance measures should be available. If there are restrictions on publicly sharing data—e.g. participant privacy or use of data from a third party—those must be specified.

Reviewer #1: Yes

Reviewer #2: Yes

**********

5. Is the manuscript presented in an intelligible fashion and written in standard English?

PLOS ONE does not copyedit accepted manuscripts, so the language in submitted articles must be clear, correct, and unambiguous. Any typographical or grammatical errors should be corrected at revision, so please note any specific errors here.

Reviewer #1: Yes

Reviewer #2: Yes

**********

6. Review Comments to the Author

Please use the space provided to explain your answers to the questions above. You may also include additional comments for the author, including concerns about dual publication, research ethics, or publication ethics. (Please upload your review as an attachment if it exceeds 20,000 characters)

Reviewer #1: • It is recommended that the introduction conclude with a clear statement of the paper's purpose, a summary of its novel contributions, and a description of its position within the specialized scientific literature.

• The statistical software should be cited as follows: GraphPad Software. (2023). GraphPad Prism (Version 9) [Computer software]. San Diego, CA: GraphPad Software, Inc. Available at https://www.graphpad.com.

• The discussion section should begin by restating the study's purpose and explicitly indicating whether this objective was achieved.

• Additionally, a subchapter titled "Study strengths, limitations, future directions, and clinical implications" should be included, or these topics should be addressed in separate subsections within the discussion section.

Reviewer #2: Manuscript ID: PONE-D-26-19080R1

Title: "Factors associated with fear and coping strategies during pandemic in female university students: The moderator role of physical activity and infection status"

General Evaluation

The authors have done an excellent job addressing the primary concerns from the first round of reviews. The clarity of the methodology, the accuracy of the baseline descriptive data representation, and the contextual framing of the timeline have all significantly improved. By switching the focus to the post-emergency phase (reflecting the July–December 2023 data collection timeline) and depositing the de-identified raw dataset as a Supporting Information file, the manuscript now complies with PLOS ONE's rigorous publication standards.

1. Major Comments and Revisions Addressed

1.1 Resolution of Sample Contradiction

1.1.1 Previous Concern: The initial draft erroneously implied that 100% of the cohort had experienced a COVID-19 infection, clashing directly with Table 1 (27.4% positive).

1.1.2 Revision Assessment: The authors clarified the text to specify that among those classified as positive, status was cross-checked with the official Turkish electronic health record system (e-Nabız). This fully resolves the previous ambiguity.

1.2 Timeline and Post-Emergency Contextualization

1.2.1 Previous Concern: The framework failed to acknowledge that data was collected in late 2023, long after the height of the acute crisis phase.

1.2.2 Revision Assessment: The text now accurately frames the study within the "post-emergency phase," adapting both the Introduction and Discussion sections to reflect long-term or residual health anxiety rather than immediate crisis behaviors.

1.3 Correction of Moderation Logic

1.3.1 Previous Concern: The explanation regarding simple slopes for recreationally active versus sedentary individuals was counterintuitive.

1.3.2 Revision Assessment: The updated Discussion provides a statistically sound interpretation. It notes that sedentary individuals' coping dynamics are highly contingent upon fear thresholds, whereas active individuals show a more stable, baseline repertoire of coping mechanisms that remain resilient irrespective of high fear.

2. Minor Observations for Final Polish

2.1 Typographical Consistency: In the Data Analysis section, the authors successfully standardized references to the dependent variable scale as COS (Coping with Outbreak Scale), eliminating the accidental "COP" shorthand found in the previous draft.

2.2Confounding Acknowledgment: The new paragraph in the Discussion section properly addresses the high bereavement rate (53.6%) among participants as a potential source of unmeasured psychological variance, providing necessary context for this uniquely high descriptive metric.

3. Recommendation

Recommendation: Accept with No Further Revisions (or minor formatting polish at the editorial level).

**********

7. PLOS authors have the option to publish the peer review history of their article (what does this mean?). If published, this will include your full peer review and any attached files.

If you choose “no”, your identity will remain anonymous but your review may still be made public.

**Do you want your identity to be public for this peer review?**  For information about this choice, including consent withdrawal, please see our Privacy Policy.

Reviewer #1: No

Reviewer #2: **Yes:** Dr Rimah Melati Ab Ghani

**********

[NOTE: If reviewer comments were submitted as an attachment file, they will be attached to this email and accessible via the submission site. Please log into your account, locate the manuscript record, and check for the action link "View Attachments". If this link does not appear, there are no attachment files.]

To ensure your figures meet our technical requirements, please review our figure guidelines: https://journals.plos.org/plosone/s/figures

You may also use PLOS’s free figure tool, NAAS, to help you prepare publication quality figures: https://journals.plos.org/plosone/s/figures#loc-tools-for-figure-preparation.

NAAS will assess whether your figures meet our technical requirements by comparing each figure against our figure specifications.

# Author comment: Author response to Decision Letter 2

**DOI:** 10.1371/journal.pone.0355544.r004

21 Jul 2026

We sincerely thank Reviewers 1 and 2 for their careful re-evaluation of our revised manuscript and for their positive feedback. We greatly appreciate their recognition of the revisions made in response to the initial comments. Their constructive suggestions have significantly improved the quality and clarity of our manuscript, and we are grateful for the time and effort they devoted to the review process.

Attachment Submitted filename: Response_to_Reviewers_auresp_2.docx

# Editor report: Decision Letter 2

**DOI:** 10.1371/journal.pone.0355544.r005

**Authors:** Wanli Zang

23 Jul 2026

Factors associated with fear and coping strategies during pandemic in female university students: The moderator role of physical activity and infection status

PONE-D-26-19080R2

Dear Dr. ERMİŞ,

We’re pleased to inform you that your manuscript has been judged scientifically suitable for publication and will be formally accepted for publication once it meets all outstanding technical requirements.

Within one week, you’ll receive an e-mail detailing the required amendments. When these have been addressed, you’ll receive a formal acceptance letter and your manuscript will be scheduled for publication.

An invoice will be generated when your article is formally accepted. Please note, if your institution has a publishing partnership with PLOS and your article meets the relevant criteria, all or part of your publication costs will be covered. Please make sure your user information is up-to-date by logging into Editorial Manager at Editorial Manager® and clicking the ‘Update My Information' link at the top of the page. For questions related to billing, please contact billing support.

If your institution or institutions have a press office, please notify them about your upcoming paper to help maximize its impact. If they’ll be preparing press materials, please inform our press team as soon as possible -- no later than 48 hours after receiving the formal acceptance. Your manuscript will remain under strict press embargo until 2 pm Eastern Time on the date of publication. For more information, please contact onepress@plos.org.

Kind regards,

Wanli Zang, Ph.D.

Guest Editor

PLOS One

Additional Editor Comments (optional):

The revised manuscript has adequately addressed the previous comments. Its overall quality has been substantially improved and now meets the journal’s publication standards. I recommend acceptance in its present form.

Reviewers' comments:

# Editor report: Acceptance letter

**DOI:** 10.1371/journal.pone.0355544.r006

**Authors:** Wanli Zang

PONE-D-26-19080R2

PLOS One

Dear Dr. ERMİŞ,

I'm pleased to inform you that your manuscript has been deemed suitable for publication in PLOS One. Congratulations! Your manuscript is now being handed over to our production team.

At this stage, our production department will prepare your paper for publication. This includes ensuring the following:

* All references, tables, and figures are properly cited

* All relevant supporting information is included in the manuscript submission,

* There are no issues that prevent the paper from being properly typeset

You will receive further instructions from the production team, including instructions on how to review your proof when it is ready. Please keep in mind that we are working through a large volume of accepted articles, so please give us a few days to review your paper and let you know the next and final steps.

Lastly, if your institution or institutions have a press office, please let them know about your upcoming paper now to help maximize its impact. If they'll be preparing press materials, please inform our press team within the next 48 hours. Your manuscript will remain under strict press embargo until 2 pm Eastern Time on the date of publication. For more information, please contact onepress@plos.org.

You will receive an invoice from PLOS for your publication fee after your manuscript has reached the completed accept phase. If you receive an email requesting payment before acceptance or for any other service, this may be a phishing scheme. Learn how to identify phishing emails and protect your accounts at https://explore.plos.org/phishing.

If we can help with anything else, please email us at customercare@plos.org.

Thank you for submitting your work to PLOS ONE and supporting open access.

Kind regards,

PLOS ONE Editorial Office Staff

on behalf of

Dr. Wanli Zang

Guest Editor

PLOS One
